# Plant–arthropod interactions of an endangered California lupine

**DOI:** 10.1002/ece3.8688

**Published:** 2022-03-08

**Authors:** Carina I. Motta, Justin C. Luong, Katja C. Seltmann

**Affiliations:** ^1^ Departamento de Biodiversidade Universidade Estadual Paulista Júlio de Mesquita Filho Rio Claro São Paulo Brazil; ^2^ Vernon and Mary Cheadle Center for Biodiversity and Ecological Restoration University of California Santa Barbara California USA; ^3^ Environmental Studies Department University of California Santa Cruz California USA

**Keywords:** coastal dune, endemic, Fabaceae, *Lupinus nipomensis*, pollination, restoration

## Abstract

The reintroduction of endangered plant species is an essential conservation tool. Reintroductions can fail to create resilient, self‐sustaining populations due to a poor understanding of environmental factors that limit or promote plant success. Biotic factors, specifically plant–arthropod interactions, have been shown to affect the establishment of endangered plant populations. *Lupinus nipomensis* (Nipomo Mesa lupine) is a state of California (California Rare Plant Rank: 1B.1) and federally (65 FR 14888) endangered endemic plant with only one extant population located along the central California coast. How arthropods positively or negatively interact with *L*. *nipomensis* is not well known and more information could aid conservation efforts. We conducted arthropod surveys of the entire *L*. *nipomensis* extant population in spring 2017. Observed arthropods present on *L*. *nipomensis* included 17 families, with a majority of individuals belonging to Thripidae. We did not detect any obvious pollinators of *L*. *nipomensis*, providing support for previous studies suggesting this lupine is capable of self‐pollinating, and observed several arthropod genera that could potentially impact the reproductive success of *L*. *nipomensis* via incidental pollination or plant predation.

## Graphical abstract

This article reports previously undocumented plant–arthropod interactions of the endangered California Nipomo mesa lupine. The presence of herbivorous arthropods and the absence of pollinators observed in this study have implications for future conservation efforts for this species.

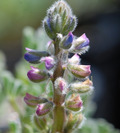

## 1 INTRODUCTION

Plant–arthropod interactions, through processes such as pollination, herbivory, and frugivory, play an important role in the reproductive success of plant species (Nemec & Bragg, 2008; Schweizer et al., 2013; Strong et al., 1995). Low pollinator visitation diversity or abundance can explain reduced distribution, low reproductive output, or failure to establish novel plant populations in habitats that are otherwise abiotically suitable (Karron, 1987; Kearns et al., 1998). Herbivorous arthropods meanwhile have been shown to negatively impact establishment and seed production of plants (Bevill et al., 1999; Münzbergová & Herben, 2005). Both the mutualistic and antagonistic interactions between plants and arthropods are important drivers in determining the survival of a plant (Stahl et al., 2018).

Despite the importance of arthropod interactions to plant species, current conservation efforts to restore endangered plants often prioritize the presence of appropriate abiotic conditions to select reintroduction sites (Falk et al., 1996; Godefroid et al., 2011; Guerrant & Kaye, 2007). Knowledge of herbivory and pollination has been shown to be important in maximizing reproduction and increasing establishment success of rare plant species (Archer & Pyke, 1991; Kay, 2008). Due to the often small population size of endangered plants, arthropod herbivores can have strong impacts through a reduction in plant size, growth, and recruitment (Ancheta & Heard, 2011; Myers & Sarfraz, 2017). Similarly, pollination is especially important to endangered plant species in order to maintain the population size and increase outcrossing of individuals (Horth, 2019; Reiter et al., 2017; Steffan‐Dewenter et al., 2001). Maintaining genetic diversity and reducing the likelihood of inbreeding depression is essential to the conservation of rare plant species (Falk, 1990; Lee et al., 2018).

**FIGURE 1:**
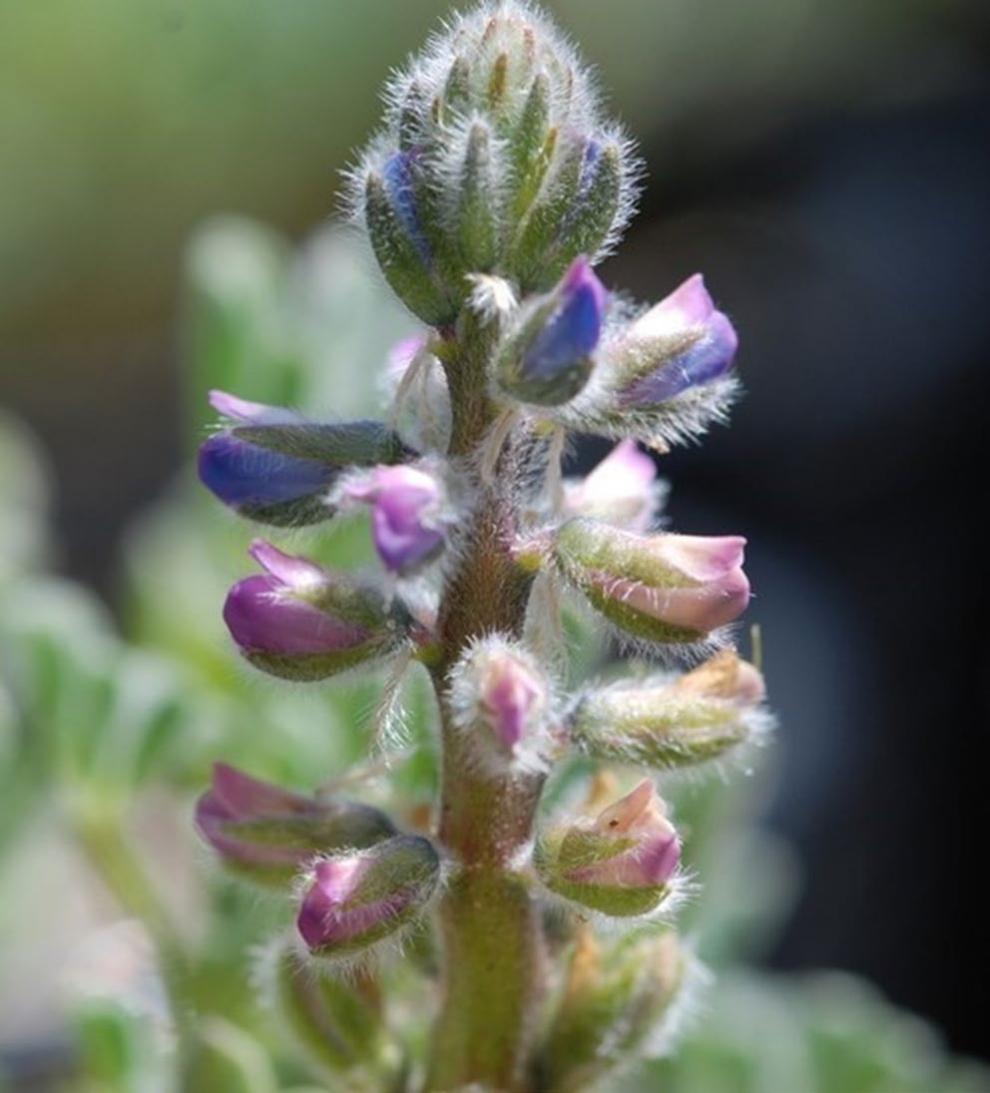
Flower peduncle of *Lupinus nipomensis* Eastw. (Nipomo mesa lupine). Image provided by JC Luong

*Lupinus nipomensis* Eastw. (Fabaceae, Nipomo Mesa lupine) is a state and federally endangered annual forb endemic to Nipomo, California, USA (USFWS, 2009; Figure 1). Historically, *L*. *nipomensis* has occurred at low densities in back dunes and inter‐dune habitat. The loss of coastal back dune habitat due to land conversion, fragmentation, and competition with the invasive perennial veldt grass (*Ehrharta calycina* Sm., Poaceae) limits the range and potential for natural regeneration of the *L*. *nipomensis* population (Skinner & Pavlik, 1994). The entire extant population is geographically isolated within a 5 km^2^ area along the central California coast in the Guadalupe‐Nipomo Dune Complex and comprises seven dispersed colonies. Total population size is dependent on winter and spring climatic conditions and ranges between 139 and 771 individuals per year (USFWS, 2019).

The reintroduction of Nipomo Mesa lupine, and other rare plants with a limited initial population size, requires specific abiotic conditions to maximize reproductive output from reintroduction efforts. Luong et al. (2019) provide an overview of abiotic microhabitat characteristics (i.e., landscape slope and aspect) and seed treatment relevant to *L*. *nipomensis* fecundity during reintroduction efforts. A foundational study of *L*. *nipomensis* by Walters and Walters (1989) primarily focuses on abiotic drivers of reproduction, specifically changes observed in flowering and fruit set as a factor of rainfall received. Walters and Walters (1989) also include a record of herbivorous arthropod interactions, but do not specifically include pollinator observations. Because other annual lupines (e.g., *Lupinus bicolor* Lindl.) are known to be pollinated by bees and flies, the absence of pollinator observations has led practitioners and researchers to hypothesize that *L*. *nipomensis* is capable of self‐pollinating (Luong et al., 2019; Moldenke, 1976; USFWS, 2019).

In this study, we sought to establish a baseline of plant–arthropod interactions of in situ *L*. *nipomensis*, especially targeted pollinator observations, which have not previously been studied. We surveyed arthropod use of *L*. *nipomensis* and classified plant visitors as potential pollinators when observed on or in the flowers. The primary goal of this study was to create an inventory of observed arthropod interactions with *L*. *nipomensis* to inform future research and conservation efforts.

## 2 METHODS

### 2.1 Study area

**FIGURE 2:**
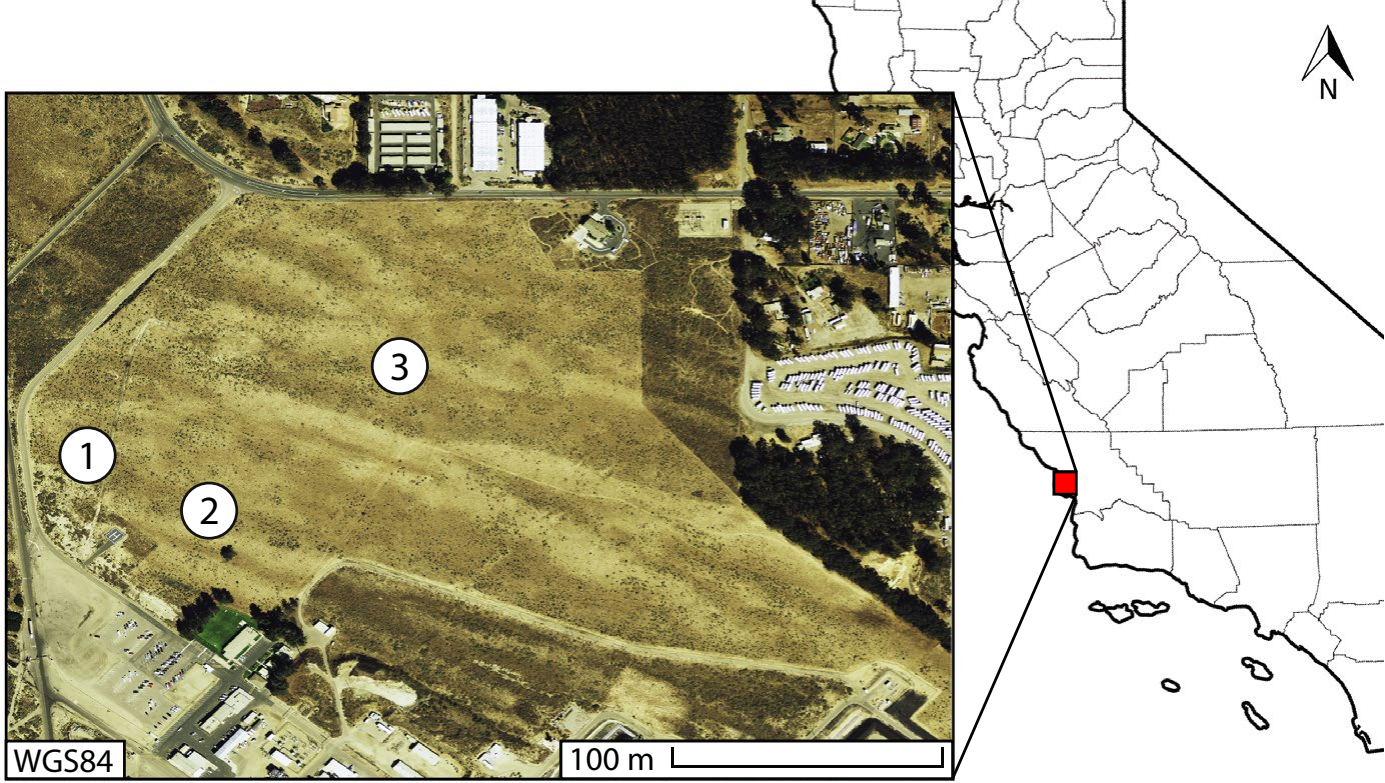
Map of sampled *Lupinus nipomensis* colonies (labeled 1–3) and geographic placement of study site in San Luis Obispo County, California, USA. The study area is indicated by the red box and county lines are displayed in gray on the map of California, USA. The inset box displaying the study area is approximately 400 m × 300 m

This study was conducted in the spring of 2017 at the Phillips 66 Oil Refinery (35.0388889, −120.5894444) in San Luis Obispo County, California, USA (Figure 2). The region is characterized by a Mediterranean‐type climate with cool, wet winters and hot, dry summers, while also receiving occasional inputs of water from coastal fog (Baguskas et al., 2016). San Luis Obispo County receives an average precipitation of 33.1 cm annually, has an average wind speed of 10.83 km/h (November–May), and average low and high temperatures ranging from 5.92 to 18.9°C during the growing season (Western Regional Climate Center, 2020). We classified cloud cover on a 4‐point scale and recorded ambient air temperature and wind speed during each visit (see complete dataset Luong et al., 2021).

Phillips 66 is required to mitigate for their oil development by establishing protected areas where extant populations are regularly monitored and restricting management actions that could negatively affect *L*. *nipomensis* populations (USFWS, 2009). This protected area in which we conducted our study is a coastal back dune ecosystem and the oldest part of a dune complex. These less disturbed areas often have later successional plants with increased soil stability as well as higher plant and insect diversity (Buckler, 1979; Ferrier et al., 2012; Miller et al., 2010). The study site is dominated by non‐native, invasive perennial veldt grass (*Ehrharta calycina*) with scattered native annual forbs and perennial shrubs. The area is actively grazed by cattle during *L*. *nipomensis* dormant season (June–November) to suppress the invasive veldt grass.

The entire population of *L*. *nipomensis* is restricted to seven colonies within the Phillips 66 protected area. Two of the colonies were excluded due to their wide and sparse distribution, making these colonies ineffective for targeted sampling. One colony was newly rediscovered from historic occurrence data and not found until partway through the course of the study. A fourth population was located along a roadside with different ambient disturbance characteristics compared to the other colonies. Plant visitor observations and vegetation monitoring were conducted at the remaining three colonies within the protected area in accordance with our California Fish and Wildlife permit (Permit No. 2081(a)‐16–010) (Figure 2). The three colonies sampled are consistent colonies containing a majority of *L*. *nipomensis* individuals and are representative of the population as a whole.

### 2.2 Plant visitor observations

One monitoring plot was established per colony (*n* = 3). Each plot was 8m × 8m and contained 22–96 *L*. *nipomensis* individuals. We conducted arthropod visitor surveys using a standardized observation‐based protocol useful for recording flower and foliage visitations (Herrera, 1990; Thompson, 2001). Observers conducted a 40‐min observation session of *L*. *nipomensis* arthropod visitors at each of the three plots per visit. A total of 48 h of *L*. *nipomensis* arthropod visitor surveys were conducted across all plots (16 h per plot) over the course of 24 visits between March 2017 and May 2017. Short observation times and single field season were due to limited accessibility, small population size, and the permitting protocol to access private property. Observations occurred between 12:00 and 15:00 (Pacific Standard Time), during the warmest part of the day when diurnal insects are most active to observe the greatest potential suite of insects visiting *L*. *nipomensis* (Willimer, 1983). One set of early and late sampling (beginning at 9:00 and 16:00, respectively) was conducted to increase the likelihood of sampling temporal niche visitors.

During each arthropod visitor survey, *L*. *nipomensis* individuals within the monitoring plot were closely observed and any visiting arthropods (floral and otherwise) were captured using aspirations and hand or net collections (Chacoff & Aizen, 2006; Kleijn & Langevelde, 2006). It is important to note that not all flower visitors observed were pollinators, but they are considered potential or “incidental pollinators.” Incidental pollinators move pollen from flower to flower while foraging for other resources (Anandhan et al., 2020; Kearns et al., 1998). Nets were only used when necessary to minimize damage as dictated by the California Department of Fish and Wildlife collection permit (Scientific Collecting Permit SC‐13574). Incidental observations of nearby arthropods visiting other plant species within the plot were also recorded, and when possible, captured. At the end of each 40‐minute survey, beat sampling was conducted on, and an inflorescence was collected from, each *L*. *nipomensis* individual in the plot. Inflorescence samples were stored in air‐tight bags with an ethyl acetate cotton ball and kept cool until returned to the lab. At the lab, inflorescences were dissected and any arthropods found were placed in 75% ethanol vials. Specimen collection complied with California State and Federal laws and samples were vouchered at the Invertebrate Zoology Collection at the University of California, Santa Barbara (specimen catalog numbers are included in Appendix S1).

Collected insects were identified to family and received lower classifications if possible (Carvalho, 1955; Daniel & Franz, 2012; Gibson et al., 1997; Herring, 1976; Hoddle et al., 2012; Iowa State University Department of Entomology, 2017; Marshall, 2012; Ross et al., 2002; Schuh & Slater, 1995; Slater & Baranowski, 1978). Specimens identified to family were sorted into putative species based on morphology or morphospecies (Samways et al., 2010). New host records were determined and recorded via the Global Biotic Interactions (GloBI) database and through literature searches (Poelen et al., 2014). Families classified as flower visitors are considered potential pollinators and were observed on or in flower parts of *L*. *nipomensis*.

### 2.3 Data visualization

Interaction data between *L*. *nipomensis* and visiting arthropods was visualized in R Studio (version 1.4.1106). An interaction web was created using the *bipartite* package (Dormann, 2021). Arthropod icons were created or retrieved from http://phylopic.org/. Icons for Asilidae, Coccinellidae, Curculionidae, and Miridae are under a creative common license (http://creativecommons.org/licenses/by/3.0/) by G Monger, M Broussard, JC Giron, and K Garcia, respectively. The map of the study area was created using WGS84 imagery from ArcGIS (version 10.4, Environmental Systems Research Institute 2012) and edited in Adobe Illustrator (version 24.1.3).

## 3 RESULTS

**FIGURE 3:**
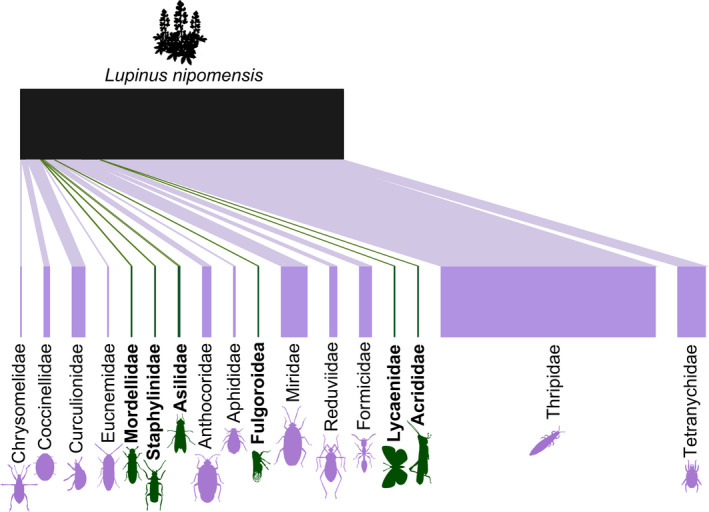
Interaction network of arthropod families containing individuals found on *Lupinus nipomensis*. Families containing at least one individual found in or on flowers are colored in purple while families only containing individuals found on other, vegetative parts of the plant are colored in green with bolded text. The figure is organized alphabetically by Order and Family within Order, corresponding to Table 1

A total of 351 arthropod individuals were observed interacting with *L*. *nipomensis* during our surveys (Table 1). Records of the 157 vouchered specimens are available on the UCSB Invertebrate Zoology Collection, Global Biodiversity Information Facility (GBIF), and Global Biotic Interactions (GloBI) databases (Appendix S1). Twenty‐two unique morphospecies from 8 orders and 17 families were classified. At least 1 individual from 11 unique families were found in or on a *L*. *nipomensis* flower (Figure 3).

**TABLE 1:** Order, Family, flower visitation, number of morphospecies, and number of individuals of arthropods that were observed interacting with *Lupinus nipomensis*

Order	Family	Flower visitor	No. of morphospecies	No. of individuals
Coleoptera				
	Chrysomelidae	Yes	1	1
	Coccinellidae	Yes	1	2
	Curculionidae	Yes	4	14
	Eucnemidae	Yes	1	1
	Mordellidae	No	1	1
	Staphylinidae	No	1	1
Diptera				
	Anthomyiidae	No	1	1
Hemiptera				
	Anthocoridae	Yes	1	9
	Aphididae	Yes	1	2
	Fulgoroidea	No	1	1
	Miridae	Yes	1	28
	Reduviidae	Yes	2	10
Hymenoptera				
	Formicidae	Yes	2	13
Lepidoptera				
	Lycaenidae	No	1	1
Orthoptera				
	Acrididae	No	1	1
Thysanoptera				
	Thripidae	Yes	1	234
Trombidiformes				
	Tetranychidae	Yes	1	31
Total			22	351

Individuals observed visiting *L*. *nipomensis* that were identified to genus and species include members of orders Diptera, Coleoptera, Hemiptera, Hymenoptera, and Lepidoptera (Appendix S1). The one Dipteran observed was *Delia lupini* (Coquillett, 1901) (Anthomyiidae). The Coleoptera species were *Diabrotica undecimpunctata* Mannerheim, 1843 (Chyrsomelidae), *Apleurus* sp., *Scaphomorphus* sp., *Trigonoscuta* sp., and *Rhigopsis effracta* LeConte, 1874 (Curculionidae). Hemipterans identified to lower classifications include *Orius* sp. (Anthocordidae), *Closterocoris amoenus* (Provancher, 1887) (Miridiae), *Lygus* sp. (Miridae), and *Apiomerus californicus* Berniker & Szerlip, 2011 (Reduviidae). Collected hymenopterans were all Formicidae, including *Crematogaster* sp. and *Linepithema humile* (Mayr, 1868). A lepidoptera individual, *Plebejus lupini* (Boisduval, 1869) (Lycaenidae), was observed landing on a vegetative part of *L*. *nipomensis*. Thysanoptera individuals were identified to genus (*Thrips* sp.) and accounted for 234 out of the total 320 individuals observed (Figure 3).

Incidental observations were made of arthropods visiting other plant species within the plot, including *Acmispon glaber* (Vogel) Brouillet (Deerweed, Fabaceae), *Amsinckia spectabilis* Fisch. & C.A. Mey. (seaside fiddlehead, Boraginaceae), *Collinsia heterophylla* Graham (purple Chinese houses, Plantaginaceae), *Ehrharta calycina* Sm. (perennial veldt grass), *Lupinus chamissonis* Eschsch. (dune bush lupine, Fabaceae), and *Nemophila menziesii* Hook. & Arn. (baby blue eyes, Boraginaceae). We observed primarily pollinating insects, such as *Apis mellifera* Linnaeus, 1758 (Apidae), *Bombus vosnesenskii* Radoszkowski, 1862 (Apidae), Syrphidae, and Lepidopterans, visiting neighboring flowers (within 0–5 m of *L*. *nipomensis*), but never visiting *L*. *nipomensis* itself. A complete list of observed individuals is available in Appendix S1.

## 4 DISCUSSION

Plant–arthropod interaction studies associated with restoration efforts are becoming more common due to a greater appreciation of the role arthropods play in the success of plants through positive (e.g., pollination) and antagonistic (e.g., herbivory) interactions (Bucharova et al., 2021; Cariveau et al., 2021; Sabatino et al., 2021). While our study did not detect obvious pollinators, we recorded a high number of arthropod individuals that could be incidental pollinators and inadvertently pollinate *L*. *nipomensis* while feeding on pollen or other plant resources (Gill, 1991). We observed over 200 Thysanoptera individuals present in *L*. *nipomensis* flowers (Table 1). Thysanoptera have been known to pollinate members of several angiosperm families, including fabaceous plants (Varatharajan et al., 2016; Velayudhan & Annadurai, 1986). Individuals of Thysanoptera we found within *L*. *nipomensis* inflorescences were observed to have pollen attached to their body (Appendix S2). However, Thysanoptera are well‐known flower pests that consume pollen, potentially causing withering of flowers and lowering plant reproductivity (Reitz, 2009). We also observed arthropods known to be important pollinators (i.e., *Apis mellifera*) visiting neighboring plants, including another lupine species, *Lupinus chamissonis* Eschsch. (Aslan et al., 2016; Hung et al., 2018). While *Apis mellifera* and other members of Apidae are known to pollinate lupine species and were detected in our survey plots, we did not observe these arthropods interacting with *L*. *nipomensis* (Luong et al., 2019). Our study supports previous findings that *L*. *nipomensis* is capable of self‐pollinating due to an apparent lack of visitation by arthropods known to pollinate, such as bees and flies (Cullen et al., 2021; Nye & Anderson, 1974; USFWS, 2019). If pollination is occurring among *L*. *nipomensis* individuals, this service is likely being performed by incidental pollinators, such as Thysanoptera.

Arthropod genera were observed that could affect the reproductive success of *L*. *nipomensis* due to herbivory. The Dipteran found, *Delia lupini* (Anthomyiidae), was collected from a gall present on a *L*. *nipomensis* individual and has been observationally implicated to reduce fecundity in a previous study of *L*. *nipomensis* (Walters & Walters, 1988). While some galls can be benign, most are detrimental to plant health and, in some cases, have been shown to threaten endangered plant species (Harris & Pitzschke, 2020; Kolesik et al., 2019). Members of Curculionidae, which are known plant pests, were observed within the stem and interacting with *D*. *lupini* galls (Johnson‐Cicalese et al., 1990; Petrova et al., 2006). The impact of *D*. *lupini* gall presence on *L*. *nipomensis* reproductivity was not quantified in this study; however, it is possible there were additional individuals we did not observe that are affecting the fecundity of this lupine. Formicidae species we observed included *Linepithena humile*, the invasive Argentine ant (Holway, 1999). Argentine ants have been shown to impact floral visitation patterns and nesting success of other arthropods with the potential to create cascading, negative effects, and reduce pollinator visitation (Plentovich et al., 2021; Sahli et al., 2016; Underwood & Fisher, 2006). The presence of a gall‐inducing and invasive arthropods may further inhibit recruitment within this singular extant population of *L*. *nipomensis*.

Plants face both biotic and abiotic barriers to reproductive success, and for rare, endangered species, these barriers can ultimately result in extirpation or extinction (Rejmánek, 2018). Small populations, like that of *L*. *nipomensis*, can face pollination limitation, as small plant populations often do not attract pollinators due to low pollen rewards (Shi et al., 2005). A reduction in pollination leads to reduced outcrossing, ultimately resulting in a lower genetic diversity that further threatens already small, rare plant populations (Gray, 2019). Simultaneously, herbivorous arthropods can impact seed production and recruitment of plant species (Lucas‐Barbosa, 2016). Active intervention may be necessary to promote outcrossing via hand pollination, as well as protect *L*. *nipomensis* from herbivorous arthropods to ensure the genetic diversity and successful establishment of new individuals (Serrano et al., 2021; Walsh et al., 2019).

Our results suggest that if pollination is occurring among *L*. *nipomensis* individuals, it is likely being performed by incidental pollinators. Additionally, we observed herbivorous arthropods that may threaten fecundity of this lupine. Further work is necessary to determine the frequency, as well as mode, of pollination and whether the potential threats this lupine faces from herbivorous arthropods will affect the establishment of novel populations during restoration efforts.

## CONFLICT OF INTEREST

Any findings or recommendations expressed in this material do not necessarily reflect the views of funding agencies. The authors declare that they have no known competing financial interests or personal relationships that could have appeared to influence the work reported in this paper.

## AUTHOR CONTRIBUTION

**Carina Isabella Motta:** Formal analysis (lead); Visualization (lead); Writing – original draft (equal); Writing – review & editing (lead). **Justin C. Luong:** Conceptualization (equal); Data curation (equal); Funding acquisition (equal); Methodology (equal); Project administration (lead); Writing – original draft (equal); Writing – review & editing (equal). **Katja C Seltmann:** Conceptualization (equal); Data curation (equal); Formal analysis (supporting); Funding acquisition (equal); Methodology (equal); Project administration (equal); Visualization (supporting); Writing – original draft (supporting); Writing – review & editing (equal).

## Supporting information

Appendix S1

Appendix S2

## Data Availability

The data that support the findings of this study are openly available in “Plant‐arthropod interactions of an endangered California lupine” at https://doi.pangaea.de/10.1594/PANGAEA.938184. Most of the data are freely available and can be used under the terms of the license mentioned on the data set description. Records of vouchered specimens can be found on the Global Biotic Interactions (GloBI) database, available at: https://github.com/globalbioticinteractions/lupinus‐nipomensis‐interactions‐2017.
